# Trends in antithrombotic drug use and adherence to non-vitamin K oral anticoagulants in the Netherlands

**DOI:** 10.1007/s11096-015-0174-4

**Published:** 2015-08-05

**Authors:** Susan Hanemaaijer, Fong Sodihardjo, Annemieke Horikx, Michel Wensing, Peter A. G. M. De Smet, Marcel L. Bouvy, Martina Teichert

**Affiliations:** Royal Dutch Pharmacists Association (KNMP), 2514JL The Hague, The Netherlands; Division of Pharmacoepidemiology and Clinical Pharmacology, Faculty of Science, Utrecht Institute of Sciences, Utrecht University, PO Box 80082, 3508 TB Utrecht, The Netherlands; Department of IQ Healthcare, Radboud Institute for Health Sciences, Radboud University Medical Centre, PO Box 9101, 6500 HB Nijmegen, The Netherlands; Department of Clinical Pharmacy, Radboud University Medical Centre, PO Box 9101, 6500 HB Nijmegen, The Netherlands

**Keywords:** Adherence, DOACs, Drug use patterns, Netherlands, NOACs, Non-vitamin K oral anticoagulants

## Abstract

*Background* Non-vitamin K oral anticoagulants (NOACs) became available in the Netherlands in 2008, providing another antithrombotic treatment besides vitamin K antagonists (VKAs) and antiplatelet agents (APAs). *Objective* To describe the patterns of antithrombotic drug use between 2008 and 2013 by examination of dispensing data form community pharmacies in the Netherlands; to determine the concomitant use of NOACs with VKAs and APAs and switching between the drug classes; and to compare adherence to NOACs with adherence to APAs. *Setting* An observational retrospective study was conducted using routinely collected dispensing data from Dutch community pharmacies. *Methods* For each calendar year, the numbers of NOAC, VKA, and APA users were calculated. Adherence was determined for NOACs and APAs by the percentage of days covered by medication (PDC). Information on the prescribed daily dose of VKAs was unavailable. *Main outcome measures* Comparison of age, sex, and co-medications of users of the three drug classes; concomitant use of different antithrombotic drug classes and switching between these in each year; and mean PDC and percentages of all users with a PDC above 80 %. *Results* NOAC use increased during the study period to 29,687 users in 2013. In that year there were 484,024 VKA users and 1313,032 APA users. Compared with users of VKAs, NOAC users were slightly younger and more frequently used antiarrhythmic drugs and beta blockers as co-medications. Substantial numbers of patients were dispensed potentially harmful combinations in 2013: 820 subjects were dispensed NOACs together with VKAs, and 684 subjects were dispensed NOACs, VKAs, and APAs concomitantly. Mean adherence to NOACs was 84.2 % compared with 87.3 % to APA. One in four NOAC users had a PDC lower than 80 % compared with one in five APA users. *Conclusion* Our findings show increasing use of NOACs by outpatients. The number of patients taking potentially harmful combinations of antithrombotic drugs was substantial. Adherence to NOACs in daily practice may be suboptimal to prevent thrombotic events.

## Impact of findings on practice

- Long-term use of NOACs increased markedly in outpatients from 2008 to 2013.
- Substantial number of patients had potentially harmful combinations of antithrombotic drugs.
- Adherence to NOACs determined from dispensing data may be suboptimal to prevent thrombotic events.

## Introduction

Vitamin K antagonists (VKA) have been used as anticoagulants for the past 70 years [1]. In the Netherlands acenocoumarol and phenprocoumon are widely used, whereas warfarin is most frequently used worldwide [2]. VKAs are highly effective in treating and preventing thromboembolic events. However, they pose difficulties in clinical practice as they have a narrow therapeutic range and delayed onset or offset of effect. Furthermore, they show a high dosage variation between individuals as well as within subjects over time. This is related to various factors such as age, body weight, genetic polymorphisms, comorbidity, co-medication, dietary vitamin K intake, and alcohol use [1, 3, 4]. Consequently VKA treatment has to be monitored closely to ensure effective anticoagulation without causing life-threatening bleeding. However, monitoring is difficult in clinical practice. A recent meta-analysis in the United States showed that patients receiving warfarin for stroke prevention were within the therapeutic range only 55 % of the time [5]. These disadvantages prompted the development of new oral anticoagulants with a rapid onset of therapeutic effect and fewer drug interactions [6, 7]. Furthermore, it is desirable that fixed doses can be used without the need for constant coagulation monitoring [1, 8–11]. Among these new non-vitamin K oral anticoagulants (NOACs), dabigatran specifically targets the coagulation enzyme thrombin, whereas rivaroxaban and apixaban inhibit factor Xa. In the Netherlands, dabigatran and rivaroxaban were licensed in 2008 for prophylaxis of venous thromboembolism in orthopaedic surgery for short-term use up to 35 days after surgery [12, 13]. When their effectiveness and safety were sufficiently proven in various randomised controlled trials [14–18], marketing approval of the NOACs was expanded in 2011 to prophylaxis for embolism related to nonvalvular atrial fibrillation [12].

An important task for pharmacovigilance is the monitoring of new drugs after their approval, including drug-related problems such as potentially harmful drug combinations and patient adherence. After NOACs were introduced as an alternative to VKAs [19], some concerns regarding their use were highlighted. First, while the effects of VKAs can be reversed by vitamin K, there is currently no specific antidote for the anticoagulant effects of NOACs [1, 10, 20]. Second, because of their short half-lives, NOACs are less effective when doses are missed [1]. Third, in the Netherlands, monitoring of VKAs is facilitated by a network of regional anticoagulation clinics that monitor about 400,000 VKA users [21], and evidence suggests this improves the time spent in the therapeutic range [5], and leads to cost savings [22, 23]. NOAC use is not monitored so closely. Fourth, although the cited advantage of NOACs is that no routine monitoring is required, knowledge of the precise anticoagulant status may be needed in patients before emergency surgery or other invasive procedures [6]. Little is known about NOAC use, their use with other antithrombotic agents and adherence [8].

### Aim of the study

The aims of this study were: to describe the patterns of use of antithrombotic drugs, including NOACs, between 2008 and 2013 by examination of dispensing data from community pharmacies in the Netherlands; to determine the concomitant use of NOACs with VKAs or antiplatelet agents (APAs) and switching between the drug classes; and to compare adherence to NOACs with adherence to APAs.

### Ethical approval

Data of pharmacists and patients were coded and anonymised prior to analysis. Use of observational data in descriptive retrospective studies in the Netherlands is not considered as an interventional trial according to Directive 2001/20/EC and to Dutch legislation, and therefore does not need to be submitted to a medical ethic committee for approval.

## Methods

### Setting

Patterns of drug use can be studied from pharmacy dispensing data [24]. In the Netherlands, the Foundation of Pharmaceutical Statistics (SFK) collected dispensing data from 95 % of the 1981 Dutch community pharmacies in 2013 [25]. SFK data provide detailed information on the drugs dispensed, including the codes from the Anatomic–Therapeutic–Chemical (ATC) system of the World Health Organization [26], the prescribed dose, and the amount dispensed. Information on patient sex and year of birth was available. The data did not provide information on clinical diagnoses. Medication of a specific patient over time was tracked within an individual pharmacy by a unique anonymous code for each patient. Information on prescribers was restricted to information on whether they were a general practitioner (GP) or any medical specialist. Drug exposure episodes were calculated by dividing the number of drugs dispensed by the prescribed dose, except for VKAs for which the dosing information was unavailable.

### Study design

We performed an observational, retrospective cross-sectional study stratified for each year between 2008 and 2013. Data of all users of a NOAC, VKA, or APA for each calendar year were included.

### Inclusion criteria

Data from all patients from Dutch community pharmacies that had provided complete dispensing data for each year and the preceding calendar year were collected. The latter information was needed to define a first dispensing, as described in the next paragraph. Patients were eligible when they received at least one dispensing of a NOAC (dabigatran, ATC code B01AE07, rivaroxaban B01AF01, or apixaban B01AF02), a VKA (phenprocoumon B01AA04 or acenocoumarol B01AA07), or an APA [acetylsalicylic acid (ASA) B01AC04, carbasalate calcium B01AC06, dipyridamole B01AC08, clopidogrel B01AC04, prasugrel B01AC22, or ticagrelor B01AC24].

### Measures

The number of NOAC users was calculated per calendar year, and stratified by short- and long-term use. Drug use periods were calculated as the percentage of days covered by medication (PDC) during a calendar year, based on dispensings in that calendar year and within the 3 preceding months. As information on diagnosis was not available, short-term and long-term antithrombotic drug use had to be distinguished in the dispensing data: subjects with a period of continuous anticoagulant use of at least 35 days for dabigatran and rivaroxaban and 38 days for apixaban were regarded as being treated long term. The remaining NOAC users were labelled as short-term users. The cutoffs of 35 and 38 days were chosen according to the guidelines for treatment duration after hip and knee surgery [13].

Patient characteristics, numbers of drug users, and proportions of co-medications and comorbidity were calculated for each antithrombotic drug class per calendar year. A first dispensing of a particular antithrombotic was defined by a dispensing without a dispensing of the corresponding antithrombotic drug class of NOACs, VKAs, or APAs during the prior 12 months. Drug cessation was defined as the absence of a refill prescription within 4 consecutive months. This threshold was chosen because in the Netherlands a first prescription is supplied for 15 days and each following prescription for 3 months. Co-medications had to be used chronically, defined as drug dispensings covering at least 91 days during a calendar year. With a normal drug supply of a maximum of 90 days per dispensing, at least two dispensings of a drug class were needed to meet this criterion. Episodes of drug use were constructed with adjustments for early refills. Co-medication for treatment of cardiovascular diseases was defined as chronic use of the following drug groups: statins (C10AA), beta-blockers (C07A), antiarrhythmic I/III (C01B), calcium channel antagonists (C08), thiazides (C03A, C03EA01), loop diuretics (C03CA01, C03CA02), renin–angiotensin–aldosterone system (RAAS) inhibitors (C09), aldosterone antagonists (C03DA), digoxin (C01AA05). Two consecutive nitrate dispensings (C01DA) were considered as a proxy for angina pectoris. Concomitant use of a loop diuretic (C03CA01 or C03CA02) and a RAAS inhibitor (C09) were taken as a proxy for heart failure. To gain a general insight into additional comorbidities, chronic medication use was mapped for blood glucose-lowering drugs (A10), thyroid drugs (H03), antidepressants (N06A), anti-dementia drugs (N06D), antipsychotics (N05), drugs for acid-related disorders (A02), drugs for asthma/chronic obstructive pulmonary disease (R03), and drugs for treatment of rheumatoid arthritis (A07EC01, L01AA01, L01CB01, L01XC02, L04AA13, L04AA27, L04AB01, L04AB02, L04AB04, L04AB05, L04AB06, L04AC03, L04AC07, L04AD01, L04AX01, L04AX03, M01CB01, M01CC01, P01BA01, P01BA02).

Concomitant use of NOACs and APAs (dual and triple antithrombotic therapy) was determined by simultaneous drug exposure for at least 91 days. Concomitant use of VKAs, for which the prescribed daily dose was not available, was defined by at least two VKA dispensings within a period of NOAC or APA use of at least 91 days. As guidelines give explicit recommendations on the use of combinations of acetylic salicylic acid (ASA) and other antithrombotic drugs, the use of these drugs was shown separately within the APA class.

Switchers from one antithrombotic drug class to another were those subjects who started the use of another antithrombotic drug class within a calendar year without further dispensing of the antithrombotic drug class used chronically previously.

Adherence to NOAC was compared with adherence to APAs. Patients with only one NOAC or APA dispensing detected in a pharmacy and without any other dispensings during 2 calendar years were excluded (‘drop-in patients’). Adherence was calculated from dispensing data as ‘drug-taking adherence’ for long-term NOAC and APA users (see definition for long-term use above). For each drug class, the PDC was calculated from the first day of drug use until the end of the calendar year. Mean percentages were compared between both drug classes. In addition, the percentages of long-term users with a PDC higher than 80 % within all long-term users were calculated for NOAC and APA users.

### Statistical analysis

**Fig. 1:**
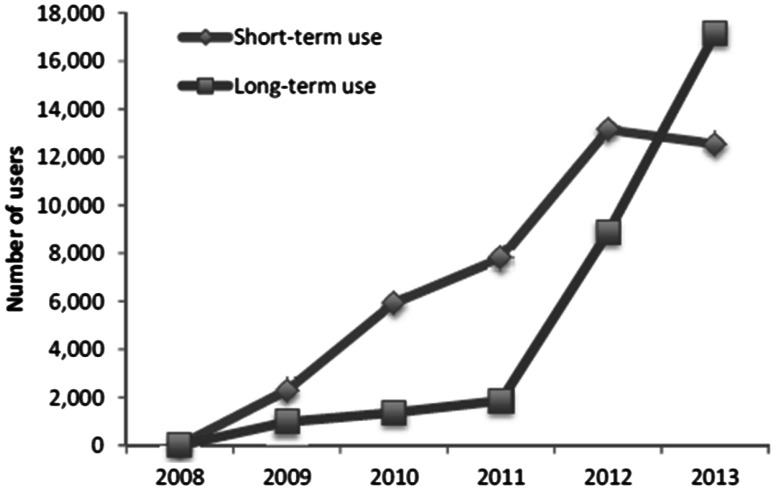
Annual increase in the total number of for short- and long-term NOAC users between 2008 and 2013. Annual numbers based on community pharmacies with data available were standardised for the total number of Dutch community pharmacies per year (see annual numbers of pharmacies with data available and the total number in the Netherlands at the bottom of Table 2)

Descriptive statistics were used for patient characteristics and the use of antithrombotic drugs and co-medications. Patient characteristics and adherence measures were compared by the Chi square test for dichotomous variables and an independent *t* test for continuous variables. A *p* value <0.05 was regarded as significant. Because of the inclusion criteria, the proportion of community pharmacies contributing data for the annual analysis varied between 73 % in 2008 to 86 % in 2013 (Table 2). In order to show the progression in the numbers of drug users between the study years for Fig. 1 and Table 2, the absolute numbers were standardised to the total number of Dutch community pharmacies in that calendar year. Statistical analysis was performed using IBM SPSS v.22.0 (IBM Corp., Armonk, NY, USA).

## Results

NOAC use increased from 18 patients in 2008 to 29,687 in 2013. Figure 1 shows the numbers of NOAC users during the study period, stratified by short- and long-term use. From 2011, the number of long-term users increased steeply and exceeded short-term users in 2013. In comparison, the total number of VKA users in 2013 was 484,024 in the Netherlands. Table 1 compares the characteristics of users of antithrombotic drug classes in 2013, based on the data provided by 1697 community pharmacies (86 % of the totally 1974 community pharmacies in the Netherlands in that year). Thus NOAC users made up 6 % of all users VKA users in 2013. NOAC users were slightly younger than VKA users (mean age 70.3 vs. 73.5 years) and used fewer co-medications, except for antiarrhythmic drugs (used in 15.7 % of long-term NOAC users compared with 7.8 % of VKA users) and beta blockers (used in 62.6 % of long-term NOAC users compared with 54.7 % in VKA users). The majority (97 %) of NOAC users were first prescribed a NOAC by a medical specialist, and 3 % by a GP.

**Table 1:** Characteristics of users of antithrombotic drug classes in 2013

	NOAC		VKA	APA
	Short-term users	Long-term users	All drug users	All drug users
Number of users	10,794	14,727	416,104	1128,782
Male (%)	4560 (42.2)	8828 (59.9)	226,965 (54.5)	633,323 (56.1)
Mean age (±SD^a^)	69.1 (±10.6)	71.2 (±10.5)	73.5 (±13.2)	70.6 (±12.5)
First dispensing (%)	9915 (91.9)	8796 (59.7)	na^b^	287,439 (25.5)
Cessation (%)	6101 (56.5)	2463 (16.7)	120,176 (28.9)	182,475 (16.2)
Medical specialist as first prescriber of drug (%)	10,403 96.4)	14,376(97.6)	na^b^	29,216 (2.6)
Cardiac co-medication				
Antiarrhythmics I/III (%)	359 (3.33)	2311 (15.7)	32,538 (7.8)	16,914 (1.5)
Statins (%)	1710 (15.8)	5455 (37.0)	163,789 (39.9)	674,613 (59.8)
Beta blockers (%)	2054 (19.0)	9218 (62.6)	227,432 (54.7)	509,047 (45.1)
Calcium channel antagonists (%)	972 (9.0)	3400 (23.1)	84,018 (20.2)	249,964 (22.1)
Thiazides (%)	711 (6.6)	1517 (10.3)	38,739 (9.3)	131,118 (11.6)
Loop diuretics (%)	394 (3.7)	2060 (14.0)	106,278 (25.5)	104,598 (9.3)
AAS inhibitors (%)	2161 (20.0)	7638 (51.9)	204,468 (49.1)	550,102 (48.7)
Concomitant use of loop diuretics and RAAS inhibitors (%)	424 (3.9)	1904 (12,9)	91,563 (22.0)	94,630 (8.4)
Aldosterone antagonists (%)	142 (1.3)	738 (5.0)	39,724 (9.6)	34,445 (3.1)
Digoxin (%)	188 (1.7)	1767 (12.0)	61,884 (14.9)	13,436 (1.2)
Two consecutive nitrate dispensings (%)	189 (1.78)	701 (4.76)	33,409 (8.03)	102,686 (9.10)
Non-cardiac co-medications				
Blood glucose lowering agents (%)	704 (6.5)	2025 (13.8)	72,517 (17.4)	214,806 (19.0)
Antipsychotics (%)	622 (5.8)	1344 (9.1)	51,665 (12.4)	133,602 (11.8)
Drugs for acid-related disorders (%)	1942 (18.0)	4911 (33.3)	147,229 (35.4)	488,212 (43.3)
Drugs for asthma or COPD (%)	724 (6.7)	1830 (12,4)	66,947 (16.1)	155,822 (13.8)
Drugs for rheumatoid arthritis (%)	108 (1,0)	172 (1.2)	6759 (1.6)	16,799 (1.5)
Antidepressants (%)	456 (4.2)	831 (5.6)	30,157 (7.3)	98,964 (8.8)
Antithyroid drugs (%)	396 (3.7)	787 (5.3)	26,372 (6.3)	63,605 (5.6)

Numbers were based on data of 1697 Dutch community pharmacies, 86 % of all Dutch community pharmacies in 2013 (The absolute numbers of NOAC users as presented in Fig. 1 and Table 2 were standardised to the total number of all Dutch community pharmacies per calendar year: 25.521 NOAC users from the 1697 community pharmacies that provided data on 2013 vs 29.687 NOAC users standardised to all 1974 Dutch community pharmacies in 2013)

Differences between all users of drug classes were compared for strata of patient characteristics, co-medications and comorbidity. Differences between numbers of NOAC, APA and VKA users were statistically significant for all parameters (*p* value < 0.01)

*NOAC* non-vitamin K oral anticoagulants, *VKA* vitamin K antagonists, *APA* antiplatelet agents, *RAAS* renin–angiotensin–aldosterone system, *COPD* chronic obstructive pulmonary disease

^a^Standard deviation

^b^Not applicable, information not available in the data

Table 2 shows the concomitant use of antithrombotic drugs and switching between drug classes per calendar year, with the absolute numbers standardized to the total number of Dutch community pharmacies in the corresponding calendar year. With the marked increase in NOAC use, the number of users with concomitant use of NOACs and other anticoagulants also increased. Although the total number was proportionately small, the number of patients was substantial: in 2013, a combination of NOACs with VKAs was dispensed to 820 subjects (2.7 % of NOAC users), while NOACs and ASA were dispensed to 446 subjects (1.5 % of NOAC users), compared with 11,207 receiving VKA and ASA (2.3 % of VKA users). Concomitant clopidogrel or prasugrel use occurred in 149 NOAC patients (0.5 % of NOAC users) compared with concomitant use in 6822 VKA patients (1.4 % of VKA users). Concomitant use of three antithrombotic drugs (NOACs with ASA and clopidogrel or prasugrel) was detected in 13 (0.05 %) patients, while 671 (0.1 %) VKA users also used two APAs concomitantly. Switching to a NOAC occurred more frequently than vice versa: 3502 subjects switched from a VKA to a NOAC, and 1552 from a NOAC to a VKA; 4850 subjects switched from an APA to a NOAC, and 2193 vice versa.

**Table 2:** Numbers of concomitant users of and switchers between antithrombotic drug classes

	2008	2009	2010	2011	2012	2013
Concomitant users of NOAC and VKA^b^	0	23	27	32.9	760	820
Concomitant users^a^ of NOAC and ASA	0	7	13	19.5	248	446
Concomitant users^a^ of NOAC and clopidogrel/prasugrel	0	0	3	6	81	149
Concomitant users^a^ of ASA and VKA^b^	11,738	12,300	12,927	12,862	12,559	11,207
Concomitant users^a^ of clopidogrel/prasugrel and VKA^b^	4737	5444	6322	7106	6730	6822
Concomitant users^a^ of NOAC, ASA and clopidogrel/prasugrel	0	0	0	0	15	13
Concomitant users^a^ of ASA, clopidogrel/prasugrel and VKA^b^	717	805	973	1088	1111	671
Switchers from NOAC to VKA	0	50	127	170	946	1552
Switchers from VKA to NOAC	0	304	120	305	3570	3502
Switchers from NOAC to APA	0	284	777	978	2077	2139
Switchers from APA to NOAC	1	456	797	1154	4078	4850
Switchers from VKA to APA	33,828	37,364	39,348	40,755	40,803	38,471
Switchers from APA to VKA	42,694	44,575	47,955	54,092	53,690	51,820
Total number of NOAC users	18	3280	7287	9662	22,009	29,687
Total number of VKA users	417,964	428,073	438,572	458,107	470,527	484,024
Total number of APA users	1263,266	1287,046	1209,941	1335,651	1324,743	1313,032
Number of Dutch community pharmacies with available data (% of all community pharmacies)	1415 (72.6)	1530 (77.4)	1557 (78.6)	1637 (82.0)	1720 (86.8)	1697 (86.0)
Total number of Dutch community pharmacies	1948 (100)	1976 (100)	1980 (100)	1997 (100)	1981 (100)	1974 (100)

Patient numbers in available data were standardised to the total number of community pharmacies per calendar year for NOAC, VKA, and APA

*ASA* acetyl salicylic acid, *PDC* percentage of days covered by medication during a calendar year

^a^Concomitant use of the drug groups for at least 91 days

^b^As the PDC could not be calculated for VKA, concomitant use with VKA is assumed for two VKA dispensings within at least 91 days of concomitant use of NOACs or APAs

In 2013, the mean drug-taking compliance for NOACs was 84.2 % compared with 87.3 % for APA (Table 3). The percentage of subjects with a PDC higher than 80 % was 75.9 % for NOACs in chronic use compared with 80.2 % for APAs.

**Table 3:** Adherence to APAs and NOACs in long term use

	APA^a^	Dabigatran^a^	Rivaroxaban^a^	Apixaban^a^
Daily use	Once	Twice	Once	Twice
Mean adherence in PDC (±SD)	**87.3** **% (21.5** **%)**	**84.9** **% (24.1** **%)**	**82.1** **% (27.7** **%)**	**92.5** **% (14.9** **%)**
Users with adherence >80% (adherent users)	830,507	7183	3535	338
Total number of users	1034,997	9473	4715	380
Percentage of adherent users within total number of users	**80.2**	**75.8**	**75.0**	**88.9**

Adherence was compared from data of the calendar year 2013

^a^Only users with a PDC > 35 days (38 days for apixaban) were included; drop-in patients were excluded. Statistically significant differences with APA are printed in bold

## Discussion

NOAC use in the Netherlands markedly increased during the study period: in 2013 there were 29,687 NOAC users compared to 484,024 VKA users. NOACs may be prescribed as an alternative for patients who did not start with VKAs because of the monitoring required, or for subjects who could not achieve stable anticoagulation with VKA treatment [27]. The latter reason is supported by the fact that switching from a VKA to a NOAC occurred more than twice as often as *vice versa*. Consistent with the guidelines, NOAC treatment was predominantly initiated by medical specialists and only occasionally by GPs. NOAC users were slightly younger and differed in the use of co-medications. With growing NOAC use, the prevalence of potentially harmful drug combinations increased: 820 subjects were detected in 2013 with concomitant use of NOACs and VKAs, a non-rational and potentially risky combination according to the guidelines [13]. Other combinations of antithrombotic drugs may have rational use for a certain duration. To examine this further, longitudinal research is needed together with information on dietary intake and diagnosis [28].

Poor adherence has been shown to be the single most important source of variations in the effects of all classes of drug treatment [29]. In clinical practice persistence with rivaroxaban was high in patients with atrial fibrillation [30]. This study showed a discontinuation rate of 15 % in the first year of treatment with rivaroxaban, mainly due to bleeding complications and other side effects. Our analysis shows that one in four NOAC users had a PDC lower than 80 % compared with one in five APA users. As we calculated adherence rates only in subjects with periods of NOAC use longer than for anticoagulation therapy in orthopaedic surgery, we aimed to address patients on lifelong NOAC therapy. Our strategy could either overestimate adherence (by falsely excluding early cessation in lifelong therapy) or underestimate it (e.g., by falsely including physician-initiated discontinuations because of major bleeding) [7]. The cut-off for adherence of a PDC of 80 % was chosen according to published studies [31]. However, this might be too low for NOACs which have short half-lives of between 7 and 17 h, and where there is a greater impact of a missed dose in achieving steady anticoagulation compared with VKAs [1, 7]. Consequently, a smaller proportion of NOAC users than VKA users may achieve effective anticoagulation in clinical practice. The impact of adherence to NOACs on clinical outcomes in daily practice is not known as yet [7, 32]. It was suggested that the advantage of NOACs in not requiring regular laboratory monitoring may translate into a lower awareness of the need to assess adherence [32]. Thus NOACs may not be suitable replacements for VKA in patients with adherence issues [1, 33]. Comparisons of adherence to VKAs and NOACs in clinical trials showed comparable poor results for both drug classes [14–16]. However, in a review of a healthcare claims database in Canada, rivaroxaban was associated with a significantly lower risk of nonadherence compared with warfarin (hazard ratio for cessation = 0.66) [34]. This could be different from the situation in Netherlands because of the therapeutic VKA monitoring by specialised anticoagulation clinics [35]. To ensure an optimal antithrombotic effect, continuous and regular intake of NOACs is necessary [32, 33]. A number of barriers to optimal NOAC use have been identified, such as a lack of knowledge of stroke risk on the one hand side and of bleeding risk on the other [36]. A combination of strategies from prescribers and pharmacists was proposed to provide tailored support for optimal use of NOACs [32, 37]. Consequently, prescribers and pharmacists should address adherence to NOACs during each patient consultation [33]. Pharmacists can contribute to better adherence [33] and have been shown to improve adherence by use of dispensing data to detect patients who did not return for their medication in time [31].

A limitation of estimating adherence from dispensing data is the lack of information on when doses were taken or missed [7]. Thus our results could overestimate adherence in daily practice, especially when the temporal sequence of the doses is essential in delivering stable anticoagulation by a drug with a short half-life of 8 to 11 h [38]. Another shortcoming of our study was the lack of information on the indication for prescribing the drugs. In order to distinguish between short- and long-term use, we used periods of drug use with cutoff points as given in the current guidelines, but this may have introduced some misclassification. However, as we used the same criteria for APAs to compare adherence, this is unlikely to have caused differential misclassification. A further shortcoming of our study was that we could not compare NOAC adherence with VKA adherence because of the lack of information on individual VKA dosages in the pharmacy data. Therefore, adherence to APAs was chosen as the best alternative for an antithrombotic drug class. The validity of dispensing data for calculating adherence measures depends on the loyalty of patients to the same pharmacy. In the Netherlands, pharmacy-shopping behaviour is low [39], and furthermore we excluded ‘drop-in’ patients. Finally, our cross-sectional study design for a comprehensive description of annual NOAC use since their introduction into the market did not allow us to evaluate the duration of antithrombotic treatment at the patient level over time to assess the duration of specific antithrombotic therapy and combinations. A strength of our study was the availability of national dispensing data, which enabled us to describe the use of antithrombotic drugs in the Netherlands in the past 5 calendar years.

## Conclusion

Our findings show a marked increase in the use of NOACs by outpatients in recent years in the Netherlands. The study indicated that a substantial number of patients require additional management to improve the safe and effective use of NOACs in clinical practice, with a reduction in non-rational combinations of antithrombotic drugs, and improved adherence.
